# Glycosyltransferase 8 domain-containing protein 1 (GLT8D1) is a UDP-dependent galactosyltransferase

**DOI:** 10.1038/s41598-023-48605-4

**Published:** 2023-12-07

**Authors:** João B. Vicente, Ana Catarina L. Guerreiro, Beatriz Felgueiras, Digantkumar Chapla, Daniel Tehrani, Kelley W. Moremen, Júlia Costa

**Affiliations:** 1 Instituto de Tecnologia Química e Biológica António Xavier, Universidade Nova de Lisboa, 2780-157 Oeiras, Portugal; 2 iBET, Instituto de Biologia Experimental e Tecnológica, Apartado 12, 2781-901 Oeiras, Portugal; 3 Complex Carbohydrate Research Center, University of Georgia, Athens, GA 30602 USA; 4 Department of Biochemistry and Molecular Biology, University of Georgia, Athens, GA 30602 USA

**Keywords:** Enzymes, Glycobiology, Biochemistry, Diseases

## Abstract

Glycosyltransferases (GTs) are enzymes that catalyze the formation of glycosidic bonds and hundreds of GTs have been identified so far in humans. Glycosyltransferase 8 domain-containing protein 1 (GLT8D1) has been associated with central nervous system diseases and cancer. However, evidence on its enzymatic properties, including its substrates, has been scarcely described. In this paper, we have produced and purified recombinant secretory GLT8D1. The enzyme was found to be *N*-glycosylated. Differential scanning fluorimetry was employed to analyze the stabilization of GLT8D1 by Mn^2+^ and nucleotides, revealing UDP as the most stabilizing nucleotide scaffold. GLT8D1 displayed glycosyltransferase activity from UDP-galactose onto *N*-acetylgalactosamine but with a low efficiency. Modeling of the structure revealed similarities with other GT-A fold enzymes in CAZy family GT8 and glycosyltransferases in other families with galactosyl-, glucosyl-, and xylosyltransferase activities, each with retaining catalytic mechanisms. Our study provides novel structural and functional insights into the properties of GLT8D1 with implications in pathological processes.

## Introduction

A wide variety of biomolecules bearing covalently bound sugar moieties attached to proteins and lipids (glycoconjugates) are found in organisms in all domains of life. Glycans on the cell surface constitute the cell glycocalyx, which plays numerous functions in cell recognition and signaling. In mammalian cells most glycosyltransferases (GTs) located along the secretory pathway, are glycosylated type II membrane proteins and contain disulfide bonds^1–3^.

Glycosylation is associated with tissue and cell homeostasis and it is dysregulated in many diseases, such as cancer or neurological diseases^4^. Alterations in the glycosylation biosynthetic machinery as consequence of mutations, expression level or localization of GTs (EC 2.4.x.y), glycosyl hydrolases, and nucleotide sugar transporters lead to dysregulated glycosylation. Indeed, mutations in glycosylation-associated genes, including GTs, are responsible for a group of rare diseases designated as congenital disorders of glycosylation, most of them affecting the central and peripheral nervous system^5^.

GTs are involved in the biosynthesis of glycoconjugates. Notably, they consist of enzymes that transfer glycosyl residues from activated sugar donors (nucleotide diphosphate sugars, nucleotide monophosphate sugars and sugar phosphates) onto nucleophilic acceptors, with the formation of glycosidic bonds. For O-GTs that use nucleotide diphosphate sugars as donors the reaction scheme is^6^:

$$[{\text{NDP}} - {\text{sugar}} + {\text{acceptor}} - {\text{OH}} \to {\text{NDP}} + {\text{acceptor}} - {\text{O}} - {\text{sugar}}]$$

During the formation of the glycosidic bond the anomeric configuration (alpha/beta) of the product can either be conserved or inverted relative to the donor substrate, and based on this, GTs are classified as either retaining or inverting enzymes, respectively^7^. GTs have been organized in families in the CAZy database (www.cazy.org) based on sequence and structural differences, and at present 116 families of GTs have been identified (as of November 6, 2023). 3-D structure analysis of the catalytic domains of GTs allowed their classification into three groups GT-A, GT-B, and GT-C based on their fold, in addition to some additional rarer fold types^8^. GT-A enzymes have one Rossman-like fold that binds nucleotides and, in general, enzymes that require metal ion for activity present a *DxD* sequence motif that coordinates a divalent metal ion and the nucleotide sugar. The GT-B group contains two Rossman-like folds and does not employ a metal ion for catalysis. GT-C GTs contain several transmembrane domains and use lipid-linked sugar donors, as found in the oligosaccharyltransferase catalytic subunit.

Recent results supported the biological importance of glycosyltransferase 8 domain-containing protein 1 (GLT8D1). Based on exome sequencing in an autosomal-dominant amyotrophic lateral sclerosis pedigree, mutations in the GLT8D1 gene were associated with the neurodegenerative disease amyotrophic lateral sclerosis^9^. The GLT8D1 gene was also found associated with psychiatric disorders such as schizophrenia^10^. Its expression was decreased in the hippocampus of schizophrenia patients and evidence indicated its involvement in the pathophysiology of the disease^11^. GLT8D1 is also associated with cancer: a mutation in GLT8D1 was found in soft tissue tumors^12^; it was proposed as prognostic marker for melanoma^13^ and gastric cancer where it correlated with tumor immunity markers^14^; it promoted human glioblastoma cell migration^15^. More recently, evidence supported a role of GLT8D1 in neurotrophin signaling within membrane lipid rafts^16^.

GLT8D1 is a type II membrane glycoprotein of 41.9 kDa (371 amino acids, UniProtKB-Q68CQ7) with three potential *N*-glycosylation sites at N103, N249 and N257. GLT8D1 is composed of a short cytoplasmic domain (amino acids 1–7), a transmembrane domain (amino acids 8–28) that anchors the protein to the membrane and a luminal part (amino acids 29–371) that includes a short stem region and a catalytic domain. GLT8D1 is localized in the trans-Golgi network of HEK293 and N2A cells^9^ and in the Golgi and endoplasmic reticulum of NCH601 cells^15^.

GLT8D1 belongs to the GT8 family of the CAZy database^17^, which includes the glycogenin glucosyltransferases (GYG1 and GYG2), the EGF domain-specific xylosyltransferases (GXYLT1, GXYLT2, XXYLT1), and the xylosyltransferase domain of the bifunctional matriglycan co-polymerase, LARGE1 and LARGE2 in *Homo sapiens* and α3-galactosyltransferase, α4-galactosyltransferase, and α-glucuronosyltransferase activities in other species. GXYLT1 and GXYLT2 were formerly known as GLT8D3 and GLT8D4, respectively; on the other hand, in mammals GLT8D1 has a closely related sequence homolog of unknown function (GLT8D2)^18^. In contrast to the glucosyl- or xylosyltransferase activity of the other mammalian GT8 enzymes, hydrolysis of UDP-galactose^9, 15, 19^ and UDP-glucose^15^ was reported for GLT8D1. So, the information concerning GLT8D1 activity and substrate specificity is still scarce.

In this work we have explored several sugar nucleotide donors and potential acceptors of recombinant GLT8D1 using differential scanning fluorimetry (DSF), a luminescent glycosyltransferase assay, HPAEC-PAD and LC–MS/MS aiming at characterizing its activity and specificity. We also examined an AlphaFold generated 3D model of GLT8D1, which provided structural insights into the protein fold, comparison with other related enzyme structures, and location of disease associated mutations.

## Results

### Production of recombinant soluble GLT8D1

**Figure 1:**
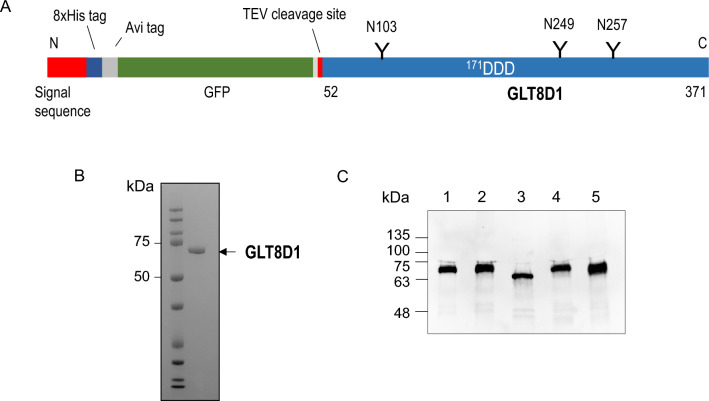
Recombinant secretory GLT8D1. (**A**) Schematic representation of the secretory GLT8D1 composed of signal sequence, His, Avi and GFP tags, TEV cleavage site and catalytic domain-containing sequence of GLT8D1. (**B**) SDS-PAGE analysis of purified GLT8D1 stained with Coomassie Blue. The amount of protein was 3.5 µg. (**C**) Deglycosylation of GLT8D1. GLT8D1 without treatment (1), or incubated with PNGase F buffer (2), PNGase F (3), Endo H buffer (4) and Endo H (5) is shown. Detection was done by the enhanced chemiluminescent method. The amount of protein applied per lane was 200 ng. Original figures (**B**) and (**C**) are presented in Fig. S7.

The GLT8D1 lumenal catalytic domain (residues 52–371) was expressed in HEK293 cells with N-terminal fusion sequences encoding a signal sequence, His_8_ tag, AviTag, GFP and TEV protease cleavage site as previously described^2^ (Fig. 1A). The recombinant secreted GLT8D1 fusion protein has an expected mass of 67,624 Da. The enzyme was purified from the culture supernatant by affinity using a Ni^2+^-NTA column with a final polishing step by gel filtration on a Superdex 75 column (Fig. S1). Purified GLT8D1 migrated as a single band in SDS-PAGE slightly below 75 kDa standard (Fig. 1B). Recovery was 3 mg of purified protein from one liter of cell supernatant.

GLT8D1 has three potential *N*-glycosylation sites. Digestion with peptide *N*-glycosidase F (PNGase F), which cleaves complex, oligomannose and hybrid N-linked glycans, caused a clear downward shift in SDS-PAGE (Fig. 1C). By contrast, with endoglycosidase H (Endo H), which cleaves only oligomannose and some hybrid glycans, there was no shift in SDS-PAGE mobility indicating that recombinant GLT8D1 is *N*-glycosylated predominantly with complex *N*-glycans.

### Screening of suitable ligands for GLT8D1 by differential scanning fluorimetry

**Figure 2:**
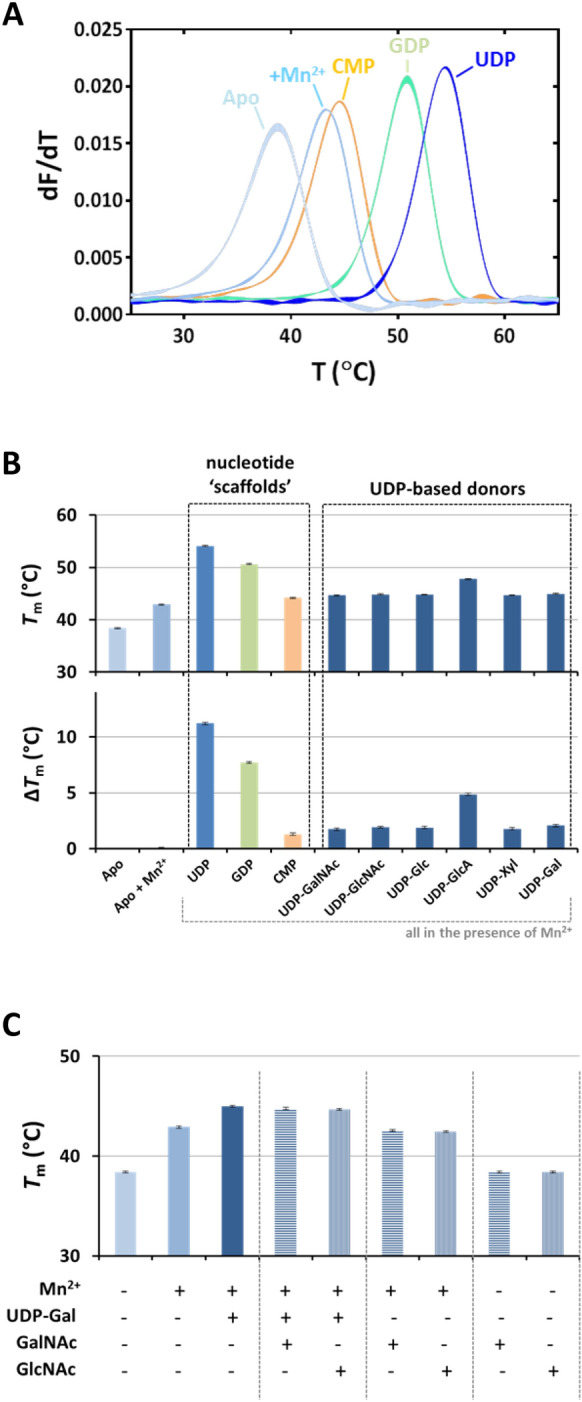
Screening of potential GLT8D1 acceptors and donors by differential scanning fluorimetry. Thermal denaturation of GLT8D1 in the presence or absence of 1 mM Mn^2+^ and/or 0.1 mM putative donors and/or 2 mM putative acceptors, monitored by intrinsic tryptophan fluorescence-based differential scanning fluorimetry. (**A**) First derivative of thermal denaturation curves obtained from the ratio of fluorescence emission at 330 nm and at 350 nm as a function of temperature (data in triplicates shown as individual curves): as isolated GLT8D1 (Apo; light blue); GLT8D1 incubated with Mn^2+^ in the absence (Apo + Mn^2+^; cyan) or presence of putative donors CMP (orange), GDP (turquoise) and UDP (dark blue). (**B**) Melting temperature (*T*_m_) values obtained by fitting the data from the ratio of fluorescence emission at 330 nm and 350 nm with a sigmoidal (variable slope) curve (data represented the average of triplicates ± standard deviation). Δ*T*_m_ values calculated by subtracting the *T*_m_ of GLT8D1 incubated with Mn^2+^ (Apo + Mn^2+^) to the *T*_m_ of GLT8D1 incubated with Mn^2+^ and putative donors (‘scaffold’ nucleotides and UDP-based donors). (**C**) Absent effect of acceptors on the *T*_m_ of GLT8D1 in the absence/presence of Mn^2+^ and UDP-Gal as donor.

Thermal denaturation of purified GLT8D1 in fusion with green fluorescent protein (GFP) monitored by intrinsic tryptophan fluorescence-based differential scanning fluorimetry (ITF-DSF) yielded a major transition with a melting temperature (*T*_*m*_) of 38.4 ± 0.1 °C (Fig. 2A) and a second minor transition assigned to GFP with a *T*_*m*_ ≈ 85 °C (data not shown). Incubation with Mn^2+^ induced a *T*_*m*_ shift of + 4.5 °C, in line with the expected stabilization afforded by Mn^2+^. From the tested ‘scaffold’ nucleotides, UDP caused the highest stabilization (Δ*T*_*m*_ =  + 11.2 °C with respect to Mn^2+^), followed by GDP (Δ*T*_*m*_ =  + 7.7 °C). CMP resulted in a modest stabilization (Δ*T*_*m*_ =  + 1.3 °C) with respect to Mn^2+^. UDP-based donors afforded lower degrees of stabilization than their UDP ‘scaffold’ (Fig. 2B). While UDP-GlcA resulted in a Δ*T*_*m*_ =  + 4.9 °C with respect to Mn^2+^, all other UDP-based donors produced Δ*T*_*m*_ values ≈ 2 °C with respect to Mn^2+^. None of the acceptors (GalNAc and GlcNAc) appeared to stabilize GLT8D1 in the presence or absence of UDP-Gal and/or Mn^2+^ (Fig. 2C).

### GLT8D1 enzymatic activity

Since the UDP nucleotide led to the highest GLT8D1 stabilization, the hydrolytic activity of the enzyme towards a panel of UDP sugar donors was investigated. Indeed, many GTs also exhibit hydrolysis activity toward their sugar nucleotide donors with water acting as an acceptor in the reaction. A panel of nine UDP monosaccharides was used in GLT8D1 assays in the absence or presence of the divalent cation Mn^2+^. As a positive control for hydrolysis, wild-type B4GalT1 and the B4GalT-Y289L mutant with additional GalNAc-transferase activity^20^ were also employed. Hydrolysis of most substrates tested with the exception of UPD-Glc and UDP-GalNAc was observed in the presence of Mn^2+^ (Fig. S2A) but the putative activity was very low, approximately 50 to 150-fold lower than the positive control with B4GalT1 (Fig. S2B). These results indicate that whereas the hydrolase activity may reflect the sugar nucleotide specificity of the enzyme as it was detected only in the presence of Mn^2+^, that activity is not efficient or selective since several donor nucleotide sugars were hydrolyzed.

**Figure 3:**
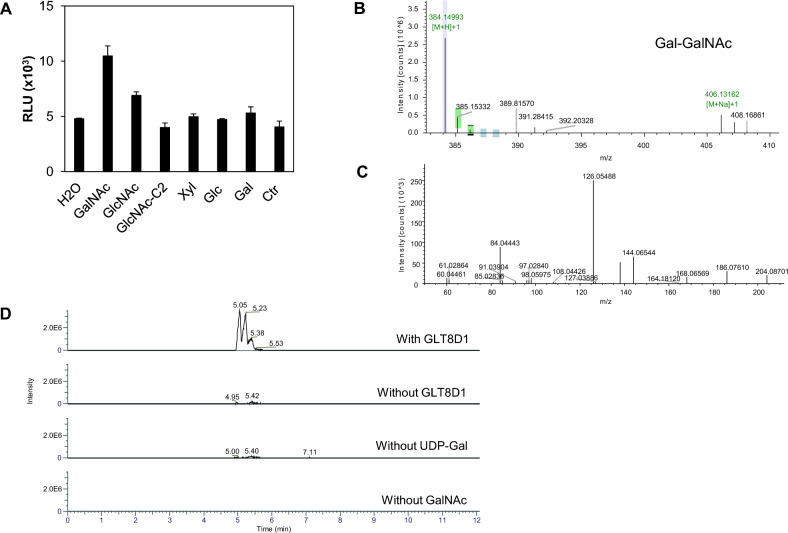
GLT8D1 activity with UDP-Gal as donor. (**A**) The assay was done as described in M&M using 0.5 mM nucleotide sugar donor, 20 mM monosaccharide, 0.8 µg GLT8D1, for 22 h. Ctr, without enzyme. Assay was done in triplicate and standard deviation is shown. (**B**) Peak at *m/z* 384.15 corresponding to the composition HexHexNAc is shown. (**C**) MS^2^ spectrum of compound at *m/z* 384.15 (see Fig. S3 for fragment identification). (**D**) Extracted ion chromatograms (XICs) of m/z 384.1500, which corresponds to the [M + H]^+^ ion of the C_14_H_25_NO_11_ compound. A 5 ppm mass tolerance was used.

Aiming at identifying potential GLT8D1 acceptors several combinations of donors and acceptors were tested using the UPD-Glo assay. With UDP-Gal as donor some transferase reaction was detected towards GalNAc and GlcNAc whereas the values with biotinylated *N*-acetylglucosamineβ-OCH_2_CH_2_NH_2_, Xyl, Glc or Gal were comparable to those of the control without enzyme (Fig. 3A). On the other hand, when UDP-GalNAc, UDP-GlcNAc or UDP-GlcA were tested as potential donors combined with GalNAc or GlcNAc as potential acceptors the release of UDP was comparable to that of the control without enzyme.

Potential products of Gal transfer from UDP-Gal onto GalNAc catalyzed by GLT8D1 were screened for by LC–MS/MS analysis. A peak at *m/z* value of 384.15 consistent with the composition HexHexNAc was detected (Fig. 3B). The extracted ion chromatogram also displayed the presence of a molecule compatible with HexHexNAc only detected when enzyme, donor and acceptor were present (Fig. 3C). In agreement a minor peak was also detected by HPAEC-PAD analysis, but the intensity was too low to allow quantification. The corresponding MS^2^ spectrum displayed fragments compatible with lacto-N-biose I and β-d-Galp-(1–3)-d-GalpNAc with FISh coverage matching of 77% (Fig. 3D; Fig. S3). Moreover, a matching of 70% with T-antigen and core 8 (α-d-Galp-(1–3)-α-d-GalpNAc) was calculated (Fig. S3). Aiming at elucidating linkage anomericity, NMR analysis was performed but the product was not detectable by this technique.

Using GlcNAc as a substrate a peak at *m/z* value of 384.15 compatible with the composition HexHexNAc was also detected (Fig. S4A). The MS^2^ spectrum of the product showed a 98.2% match with that of standard *N*-acetyllactosamine (Galβ4GlcNAc, LacNAc) analyzed in the same conditions (Fig. S4B). In agreement, a 97.4% match with LacNAc was obtained using mzCloud search.

A number of other compounds (Tables S1, S2) were assessed but found not to react as acceptors (see Figs. S5, S6 and related discussion).

### Structural modeling of GLT8D1

**Figure 4:**
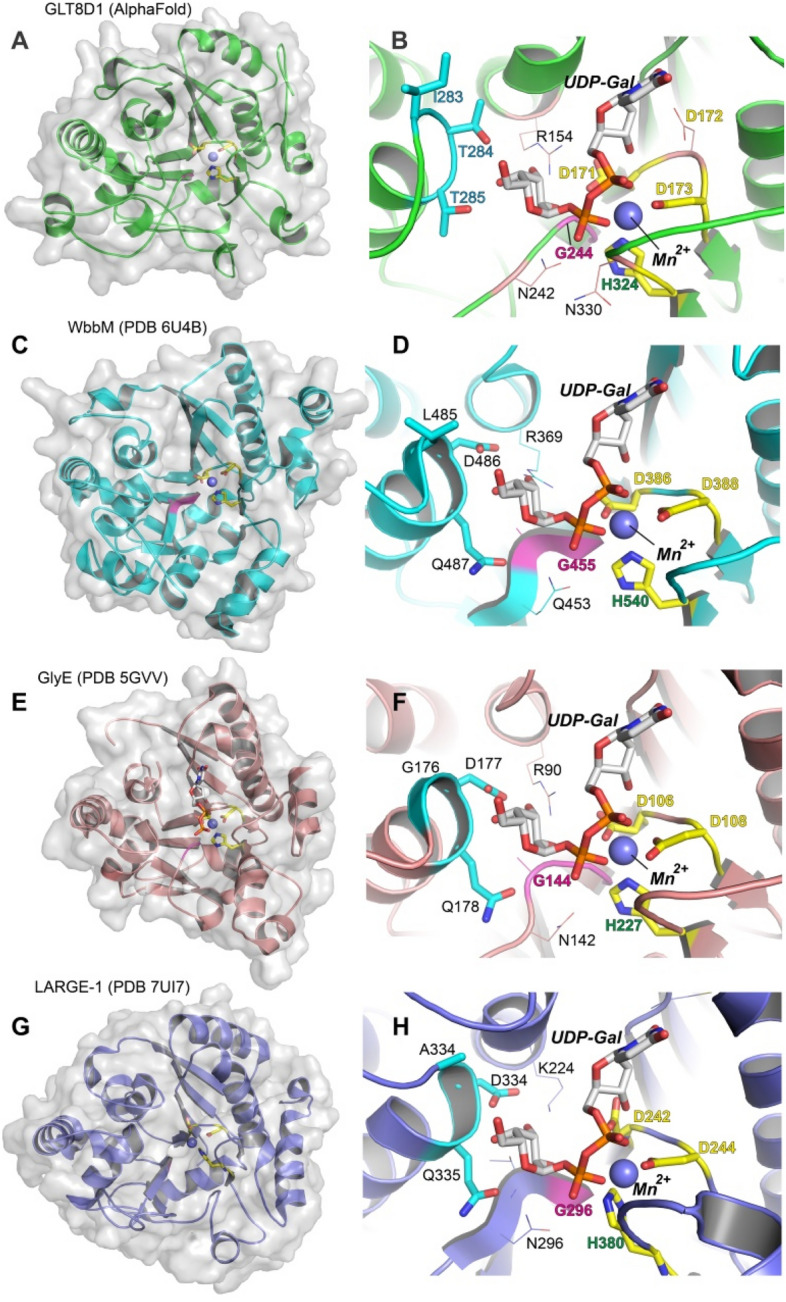
Comparison of the AlphaFold model of GLT8D1 with other related GT8 enzymes. The GT-A fold catalytic domain from the GLT8D1 AlphaFold model (AF-Q68CQ7-F1, **A** and **B**) was aligned with the related GT8 enzymes, WbbM (panels **C** and **D**, PDB 6U4B^23^), GlyE (panels **E** and **F**, PDB 5GVV^24^), and the xyloxyltransferase domain of human LARGE-1 (panels **G** and **H**, PDB 7UI7^25^). Panels **A**, **C**, **E** and **G** are displayed as cartoon representations with gray surface and stick representations for the *DxD*, G-loop and C-terminal His motifs shown in yellow and bound divalent cation as gray sphere. Panels B, D, F and H are zoomed-in representations of the donor binding site for the respective enzymes. For the zoomed-in representations, a bound UDP-Gal and Mn^2+^ were modeled in the active site (gray stick representation) based on the UDP and Mn^2+^ complex of GlyE and the structure of UDP-Gal from B4GALT1^26^ (PDB 2FYC). Putative interacting residues were identified in the respective complexes as residues facing the sugar residue of the donor. Yellow sticks are displayed for the DxD motif (yellow labels) and C-terminal His (green labels). The G-loop motif is displayed in magenta and the xED motif residues are displayed in cyan sticks in each panel. Other interacting residues are indicated by thin stick representation with residue labeling.

**Figure 5:**
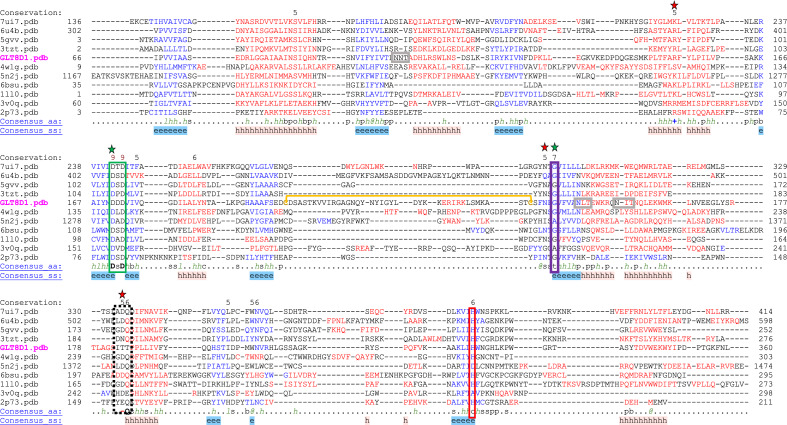
Alignment of structures similar to GLT8D1 using the PROMALS3D server. The full length AlphaFold model of GLT8D1 was used to search the Dali^27^ database (Fig. 4) and the structures of the top ten scoring proteins were aligned with the AlphaFold structure of GLT8D1(UniProt Q68CQ7) and structures of *Klebsiella pneumoniae* WbbM (PDB 6U4B), *Streptococcus pneumoniae* GlyE (PDB 5GVV), *Homo sapiens* LARGE-1 (PDB 7UI7), *Chaetomium thermophilum* UGGT (PDB 5N2J), *Anaerococcus prevotii* GT8 protein (PDB 3TXZT), *Oryctolagus cuniculus* Glycogenin-1 (PDB 1LLO), *Mus musculus* XXYLT1 (PDB 4WLG), *Homo sapiens* ABO (PDB 3V0Q), *Chlorella* virus UDP-Glc GlcT (PDB 2P73), and *Arabidopsis thaliana* XXT1 (PDB 6BSU) using PROMALS 3D^28^. Conserved secondary structure elements are indicated as helices (h) or beta strands (e) and consensus amino acid positions are classified by amino acid character (aliphatic (l), aromatic (@), hydrophobic (h), alcohol (o), polar (p), tiny (t), small (s), bulky (b), positively charged (+), negatively charged (−), and charged (c)) or as bold uppercase for conserved. Landmark GT-A fold protein motifs include the DxD motif (green box), G-loop (purple box), divergent sequences of the xED motif that are not conserved in retaining GT8 enzymes (black dotted box, and C-term His (red box). The single disulfide bond predicted in the GLT8D1 structure is indicated by the yellow bracket. Three Asn-linked glycan sequons in GLT8D1 are indicated by gray boxes.

Prior studies have investigated the modeling of the GLT8D1 protein structure and residues potentially involved in substrate interactions^15^. Recent developments of structural modeling using AlphaFold2^21^ provided the generation of an alternative model for GLT8D1 here that allows comparisons with other known related GTs (Fig. 4). As expected for a member of the GT8 family, the AlphaFold2 model of GLT8D1 contained the landmark features of a GT-A fold enzyme, including a DxD motif, G-loop, and C-terminal His residue in the sugar nucleotide donor binding site (Figs. 4A,B and 5)^22^. The fourth landmark feature of GT-A fold inverting enzymes (the ‘xED’ motif that harbors the catalytic base) was comprised of residues I283–T284–T285, not consistent with its use as a catalytic base or inverting catalytic mechanism, but consistent with the predicted retaining catalytic mechanism for GT8 enzymes^6, 22^.

Comparison of the GLT8D1 AlphaFold2 model with other known protein structures using Dali^27^ allowed the identification of several glycosyltransferases from CAZy GT8 (Table S3, Fig. 5) which use: UDP-Gal as sugar donor (bacterial WbbM^23^ and GlyE^24^); UDP-Xyl as donor (human LARGE-1^25^ and XXYLT1^29^); UDP-Glc as donor (glycogenin^30^); or a bacterial enzyme of unknown specificity (PDB 3TZT). Additional related structures were identified in the Dali analysis: GT24 fungal UGGT^31^ that uses UDP-Glc as donor; GT6 human ABO^32^ that uses UDP-Gal/GalNAc as donor; and two enzymes from GT34 that employ UDP-Glc^33^ or UDP-Xyl^34^ as donors. These results are also consistent with a broad collection of GT8 subfamily members, as well as members of GT24, GT34, GT6 and GT88 that all partition into a common clade (Clade 9) and employ a retaining glycosyltransferase catalytic mechanism^22^.

While most of the structures related to GLT8D1 in the Dali search contain bound UDP or divalent metal, few contain an intact sugar nucleotide donor (only glycogenin^30^ and ABO^35^). Preliminary modeling of UDP-Gal and Mn^2+^ in the active site of GLT8D1 could be approximated using alignment with related enzymes with bound donor substrates, including the UDP and Mn^2+^ complex of the closely related *S. pneumoniae* GlyE^24^ (PDB 5GVV) and the structure of UDP-Gal from B4GALT1^26^ (PDB 2FYC) (Fig. 4). While this model approximates the position of the bound donor complex for GLT8D1 similar to prior modeling studies^15^, it also allows a comparison of similarly positioned residues in the other related enzymes (Fig. 5). Residues facing the sugar residue of the donor in the GLT8D1 model include R154, D171, G244, N242, and T284 (Fig. 4A,B). Of these, D171 and G244 are within the DxD motif and G-loop, respectively, and are highly conserved among the 10 related enzymes (Fig. 5). The equivalent of R154 was a Lys or Arg residues in all related enzymes except the xylosyltransferase, XXYLT1, where a Ser is found in that position. The equivalent of N242 was an Asn or Gln for all related enzymes except for the glucosyltransferase, UGGT, that contains an Ile and the human Gal/GalNAc transferase, ABO, that contains a Gly in the equivalent positions. The equivalent of T284 is generally an Asp or Asn for the related enzymes except for the glucosyltransferase, glycogenin (substituted with a Gly), and *Chlorella* glucosyltransferase (substituted with a Glu). Thus, there is no apparent correlation between the donor specificity of the respective GLT8D1-related enzymes and the residues that face toward the sugar in the UDP-sugar donor binding subsites.

## Discussion

In this study, we investigated catalytic and structural properties of recombinant soluble glycosyltransferase GLT8D1. Our results indicated that the enzyme has preference for UDP nucleotide sugar donors and shows galactosyltransferase activity preferentially towards monosaccharide GalNAc as acceptor. A 3D model of GLT8D1 pinpointed structural features of GT-A fold glycosyltransferases such as the divalent metal and nucleotide donor binding sites with features consistent with a predicted retaining catalytic mechanism.

Recombinant GLT8D1 was produced as a truncated soluble secreted catalytic domain fusion protein in HEK293 cells as previously described for the expression and purification of mammalian glycosyltransferases^2^. The secreted product was *N*-glycosylated based on SDS-PAGE mobility shift following PNGase F digestion as predicted by the presence of 3 *N*-glycan acceptor sequons. Prior studies had identified a glycan structure on N249 of GLT8D1 from human liver using a combination of enzyme digestion and hydrazide chemistry^36^.

Differential scanning fluorimetry using ITF-DSF showed a high sensitivity binding of Mn^2+^ and putative donors to GLT8D1 likely due to the fact that the enzyme has 8 tryptophan residues, 3 of which are quite close to the active site. Stabilization by Mn^2+^ agrees with a GT-A fold structure for GLT8D1 and also in line with the hydrolysis results in the absence or presence of the cation. Nucleotide stabilization of GLT8D1 was greatest with UDP, followed by GDP, while CMP had almost no effect, suggesting that UDP sugars were preferred donor substrates. However, among all the UDP-monosaccharides tested, UDP-GlcA caused the highest GLT8D1 stabilization, which did not correspond to a higher hydrolytic activity in our assays; curiously, some members of the GT8 CAZy family have α-glucuronosyltransferase activity. Overall, ITF-DSF has utility to investigate potential substrates for glycosyltransferases. In agreement, others had already reported on the use of conventional DSF with SYPRO Orange fluorescent dye to study the binding of sugar nucleotides to bacterial glycosyltransferases^37^.

Many GTs are capable of hydrolyzing their sugar nucleotide donors in the absence of any acceptor, which is particularly useful to characterize activities when the acceptor substrates are unknown^38, 39^. Here, GLT8D1 appeared to hydrolyze UDP-Gal, UDP-Xyl, UDP-GalNAc, UDP-GlcA, UDP-GalA, UDP-Arap and UDP-Araf in the presence of Mn^2+^, but the activity values were low compared with controls of well-described glycosyltransferases B4GALT1 and B4GALT1-Y289L. Some GTs are acceptor-dependent enzymes, such as, fucosyltransferase 7^39^, which could be the case of GLT8D1, and only assays in the presence of the proper acceptor substrate would demonstrate activity. Other reports from the literature showed UDP-Gal hydrolysis by full-length GLT8D1^9, 15^, and also UDP-Glc hydrolysis^15^ but the activity values also appear low. It is not known how the presence of the cytoplasmic/transmembrane/stem region from the full-length form, absent from our recombinant secretory form, could affect enzyme specificity. For example, secretory fucosyltransferases 3 and 5 had increased activity toward glycoproteins but decreased activity with glycosphingolipids, compared to the full-length enzymes^40, 41^.

GLT8D1 galactosyltransferase activity was investigated using UDP-Gal as donor with several acceptors. We found that the enzyme catalyzed the transfer in vitro of Gal onto GalNAc with the formation of the disaccharide HexHexNAc as detected by LC–MS. The product was consistent with Galβ3GalNAc (lacto-N-biose I and T-antigen or core 1 O-GalNAc glycan) and Galα3GalNAc (core 8 O-GalNAc glycan) as concluded from MS/MS analysis. Since members of the CAZy GT8 family have been shown to employ retaining catalytic mechanism^22^, it is anticipated that the most likely enzymatic product would be Galα3GalNAc. Similarly, a disaccharide compatible with LacNAc structure (HexHexNAc) was detected by MS with GlcNAc as an acceptor. However, no activity was detected towards truncated *N*-glycans with terminal GlcNAc possibly due to the low concentration used and because such compounds are not efficient substrates.

In view of the low galactosyltransferase reaction efficiency observed with GalNAc as acceptor, it was concluded that this monosaccharide is not the ideal GLT8D1 substrate. However, glycans of higher complexity from carrier molecules in specific structural settings may be better substrates. Potential candidates could be glycolipids, which are known to be relevant in signaling events associated with neurodegeneration^42^ and cancer^43^. In this context, recent evidence indicated that GLT8D1 was relevant in ganglioside biosynthesis^16^. Glycoproteins could also constitute potential substrates; for example, the T-antigen and related structures are present in mucins with important roles in cancer^44^ and sialylated T-antigen is abundantly expressed in brain tissue glycoproteins^45–47^, but the enzymes involved in their biosynthesis have been well-studied. On the other hand, core 8 is a low abundance structure in glycoproteins from brain tissue^46, 48^ with unknown functional relevance, and GLT8D1 appears to have the capacity to synthesize that structure. In view of the several open possibilities, it is necessary to investigate further to find efficient endogenous GLT8D1 substrates, to structurally characterize them and evaluate their role in the mechanisms underlying diseases of the central nervous system and cancer.

Prior efforts at modeling the GLT8D1 structure revealed a GT-A fold for the enzyme^49^, including conserved landmark features^15^, and the absence of an xED motif sequence that harbors the catalytic base in inverting enzymes that is consistent with the retaining mechanism for the GT8 family enzymes^22^. We employed an AlphaFold2 model of GLT8D1 to query structure databases and identify well-characterized enzymes with high similarity to GLT8D1. Numerous GT8 proteins were identified as well as structures from GT24, GT6, and GT34 as anticipated from prior informatic analyses that clusters these families into a broader Clade 9^22^. Among the mammalian GT8 enzymes, GLT8D1 has closest sequence homology to GLT8D2, a protein of unknown function, while other mammalian members of CAZy GT8 include the glycogenins (GYG1 and GYG2), members of a xylosyltransferase family of enzymes that modify EGF-like domains (GXYLT1, GXYLT2, XXYLT1) and the xylosyltransferase domains of LARGE1 and LARGE2, all of which are more distantly related to GLT8D1/GLT8D2.

**Figure 6:**
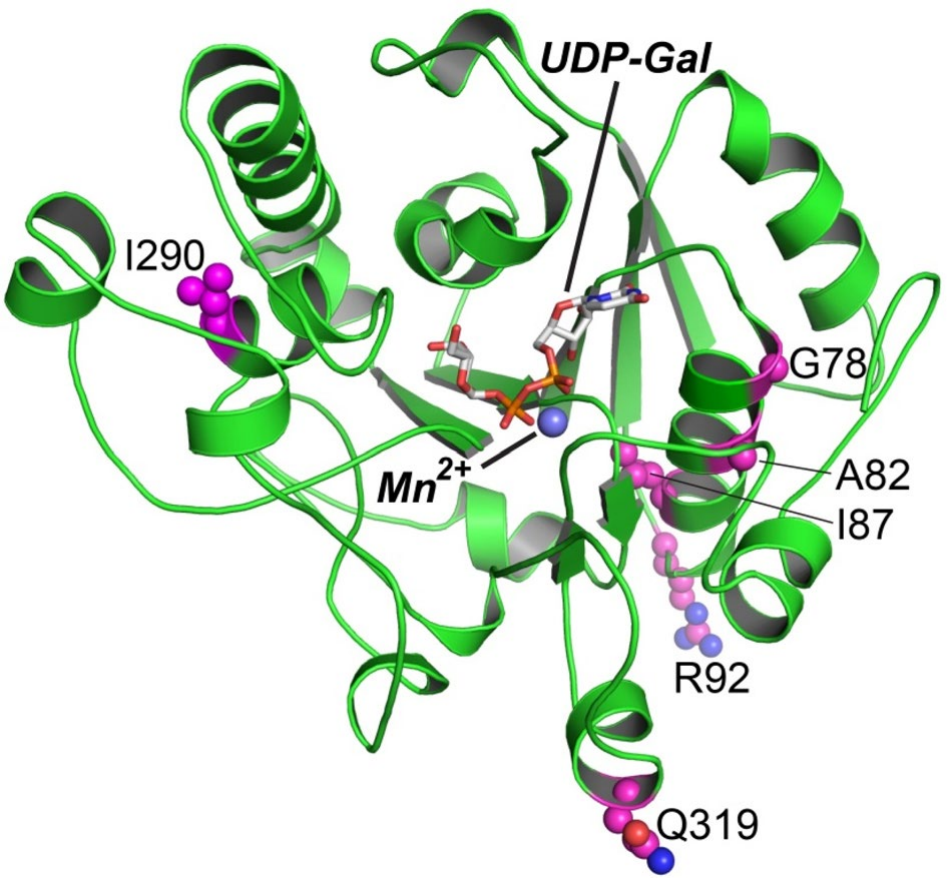
Mutations related to ALS or soft tissue tumors. Residues in the GLT8D1 catalytic domain associated ALS (G78W, A82E, I87N and R92C, I290M) or soft tissue tumors (Q319E) are displayed as magenta spheres within the cartoon representation of GLT8D1. The position of bound UDP-Gal and Mn^2+^ modeled in the GLT8D1 active site are shown in gray stick and slate sphere representation, respectively as shown in Fig. 4B.

Several GLT8D1 mutations have been identified in ALS patients including G78W, I70T, A82E, I87N, R92C 9 and I290M^9^; decreased galactosyltransferase activity was reported for G78W, R92C and I290M mutant. On the other hand, the Q319E substitution was found in soft tissue tumors^12^. Each of these mutations were located quite distant from the putative enzyme active site (Fig. 6) and likely either result in enzyme destabilization or possibly alteration of interactions with other proteins.

In conclusion, we have obtained purified, secreted form of GLT8D1 suitable for enzymatic and structural studies. Our results support that GLT8D1 exhibits low galactosyltransferase activity, but more studies are required to unveil efficient substrates, particularly endogenous substrates that would clarify the functional role of GLT8D1. GLT8D1 was modeled as a GT-A fold structure with conserved active site features in comparison with other related enzyme structures, and disease-associated mutations were mapped on the structural model, but will require future study to identify the structural basis of pathogenesis.

## Materials and methods

### Enzyme production and purification

A fusion protein expression construct encoding the catalytic domain of human GLT8D1 (Uniprot Q68CQ7, residues 52–371) was generated by PCR amplification of the GLT8D1 coding region from a Mammalian Gene Collection clone followed by Gateway BP recombination into the pDONR221 vector and subsequent transfer to the pGEn2 expression vector by Gateway LR recombination^2^. The resulting GLT8D1-pGEn2 expression construct encodes a 25-amino acid signal sequence, an His_8_ tag, AviTag, the “superfolder” GFP coding region, and the 7-amino acid recognition sequence of the tobacco etch virus (TEV) protease followed by the human GLT8D1 catalytic domain. The recombinant human GLT8D1 fusion protein was expressed as a soluble secreted product by transient transfection of suspension culture HEK293-F cells (FreeStyle™ 293-Fcells, Thermo Fisher Scientific, Waltham MA) as previously described^50–52^. The recombinant protein was purified using Ni–NTA Superflow (Qiagen, Valencia, CA) chromatography as previously described^2^ and was concentrated to approximately 3 mg/mL using an ultrafiltration pressure cell (Millipore, Billerica, MA) with a 10-kDa molecular mass cutoff membrane. The recombinant human GLT8D1 was further purified by a Superdex 75 chromatography (GE Healthcare) preconditioned with a buffer containing 20 mM HEPES, 150 mM NaCl, 0.05% sodium azide, pH 7.0. Peak fractions were pooled, concentrated to 1 mg/mL and buffer exchanged with 20 mM HEPES, 100 mM NaCl, 0.05% sodium azide, pH 7.0, 10% glycerol. The final protein preparation was aliquoted and stored at − 80 °C until use.

### Immunoblotting analysis and protein deglycosylation

Proteins were analyzed by SDS-PAGE and transferred to polyvinyledene fluoride membranes that were blocked for 1 h with 5% skimmed dry milk (Nestle) in Tris-buffered saline with 0.1% Tween-20. Primary and secondary antibodies were mouse anti-His Tag IgG1 monoclonal (1:7000; GenScript A00186) and sheep anti-mouse IgG coupled to HRP (1:3000; Amersham, GE Healthcare Europe GmbH, NA934). Washings were with TBST. Detection was performed with the Immobilon Western Chemiluminescent HRP substrate (Millipore). GLT8D1 was digested with peptide *N*-glycosidase F (PNGase F, Roche) and endoglycosidase H (Endo H, Roche), as previously described^53^, and further analyzed by immunoblotting.

### Differential scanning fluorimetry

ITF-DSF assays were performed in a Prometheus NT48 (NanoTemper Technologies GmbH, Munich, Germany). GLT8D1 at 350 µg/mL, in 50 mM TrisHCl pH 7.5, was pre-incubated with 0.1 mM of nucleotides or putative donors (UDP, GDP, CMP, UDP-Gal, UDP-Glc, UDP-Xyl, UDP-GlcA, UDP-GalNAc, UDP-GlcNAc) and/or 2 mM of putative acceptors (GalNAc and GlcNAc), in the absence or presence of 1 mM MnCl_2_, and transferred into 10 μL optical capillaries (NanoTemper). A linear temperature gradient (1 °C/min) between 20 and 90 °C was applied, and protein unfolding was monitored by recording the protein intrinsic tryptophan fluorescence emission at 330 nm and 350 nm (fluorescence excitation at 275 nm). Data in triplicates from the ratio of fluorescence emission at 330 nm and 350 nm were fitted with GraphPad 6, employing a sigmoidal (variable slope) curve and the melting temperatures (*T*_*m*_) were retrieved from the inflexion points.

### Enzyme activity assays

UDP-monosaccharide donors tested were UDP-galactose (UDP-Gal, Promega, V7171), UDP-*N*-acetylglucosamine (UDP-GlcNAc, Promega, V7071), UDP-*N*-acetylgalactosamine (UDP-GalNAc, Promega, V7081), UDP-glucose (UDP-Glc, Promega, V7091), UDP-glucuronic acid (UDP-GlcA, Promega, V7321); UDP-xylose (UDP-Xyl), UDP-arabinopyranose (UDP-Arap), UDP-arabinofuranose (UDP-Araf) and UDP-galacturonic acid (UDP-GalA) (CarboSource Services, Complex Carbohydrate Research Center, University of Georgia).

Acceptor monosaccharides included galactose (Gal), *N*-acetylgalactosamine (GalNAc), *N*-acetylglucosamine (GlcNAc), xylose (Xyl), glucose (Glc), biotinylated GlcNAcβ-OCH_2_CH_2_NH_2_ (GlcNAc-C2), di-, tri-, tetra- and pentasaccharides (Table S1) and *N*-glycans (Table S2).

Nucleotide sugar hydrolysis assays were performed in 20 μL reactions containing 200 μM UDP-sugar substrates and 10 μL (3 μg) of GLT8D1 enzyme containing 10 mM MnCl_2_, 1 mg/mL bovine serum albumin, 25 mM HEPES, 300 mM NaCl, pH 7.5. Reactions were carried out for 24 h at 37 °C and the resulting UDP was quantified using a UDP-Glo Assay (Promega Corporation, V6971) and compared to a UDP standard curve to calculate the hydrolysis activity.

For sugar transferase assays, reaction mixtures contained 50 mM Tris–HCl, pH 7.5, 5 mM MnCl_2_, 0.1 mM ultrapure UDP-monosaccharide donor, acceptors, GLT8D1 enzyme; typically, 2 μg of enzyme were used in 60 μL of reaction mixture from which 25 μL were for the UDP-Glo Assay. Acceptor monosaccharides, GlcNAc-C2 and *N*-glycans were tested at 20 mM, 1 mg/mL and 20 µM concentrations, respectively.

### HPAEC-PAD analysis

For desalting, *N*-glycan solutions (typically 0.8 mL) were applied onto Hypercarb cartridges (25 mg; Thermo Fisher 60106-304), pre-equilibrated with 0.8 mL 80% acetonitrile 0.1% TFA and washed with 3 × 0.8 mL water as described before^54^. *N*-Glycans were bound and cartridges were washed with 3 × 0.8 mL of 25 mM ammonium hydrogen carbonate and 3 × 0.8 mL of water; elution was with 0.8 mL 40% acetonitrile 0.1%TFA. The eluate was neutralized with 2.5% ammonia, dried in the Speed Vac and further analyzed by high-performance anion exchange chromatography with pulsed amperometric detection (HPAEC-PAD) or mass spectrometry (MS).

HPAEC-PAD analysis of *N*-glycans was performed using an ICS-3000 ion chromatography system (Dionex Corporation), consisting of an AS autosampler, a DC detector/chromatography module and a DP dual pump essentially as described before^53^. Twenty microlitre samples were injected into a CarboPac PA200 column (3 × 250 mm, Dionex). Released oligosaccharides were eluted with a gradient of 0.075 M sodium hydroxide (solvent A) and 0.075 M sodium hydroxide/0.6 M sodium acetate (solvent B), at a flow rate of 0.4 mL/min. The gradient consisted of a 3 min isocratic run at 100% solvent A, followed by consecutive increases of solvent B concentration (7 min to 0.7%, 15 min to 5%, 11 min to 16%, 9 min to 30%, 2 min to 100%) and a final step of 13 min at 100% B. The column temperature was at 30 °C. The electrochemical detection of the oligosaccharides was performed by application of the detection potentials and durations as recommended by the manufacturer.

### Mass spectrometry analysis

Desalted glycans were analyzed in a QExactive Focus Orbitrap (Thermo Fisher Scientific, MA, USA) coupled to a Dionex Ultimate 3000 UHPLC (Ultra-High Performance Liquid Chromatography). The separation was achieved in a Thermo Hypercarb column (2.1 × 100 mm, 3 µm particle size). A gradient of 0.1% formic acid in water (A) and 0.1% formic acid in acetonitrile (B) was applied as follows: 0–13 min, 1–99% B; 13–15 min, 99% B; 15–16 min, 99–1% B; 16–20 min, 1% B. The flow rate was 0.4 mL/min and the column temperature was kept at 30 °C. The eluate was infused into the MS through a heated electrospray (HESI) source. The parameters used were: sheath gas = 60 arbitrary units (a.u.); aux gas = 20 a.u.; sweep gas = 0 a.u.; spray voltage = 3 kV, capillary temp. = 320 °C; aux. gas heater temp. = 320 °C. To facilitate compound identification, a data-dependent MS/MS acquisition method was performed, where the top 3 most intense ions were selected for higher-energy collisional dissociation (HCD). This method consisted of several cycles of Full MS scans (R = 70,000 full width at half maximum (FWHM) at m/z 200; Scan range = 75–1125 m/z; 1 × 106 automatic gain control (AGC)), followed by 3 ddMS2 scans (R = 17,500 FWHM at m/z 200; collision energy (CE) = 30 eV; 1 × 105 AGC; maximum injection time (IT) = 100 ms; dynamic exclusion = 6 s) in positive mode. Internal calibration was performed during acquisition using Lock mass of 144.98215 m/z and 445.12003 m/z (positive).

Raw data generated in the untargeted analysis were processed using Compound Discoverer 3.2 (version 3.2.0.421, Thermo Scientific) for metabolite identification (including unsupervised peak detection). The identification searches were performed against the mzCloud MS/MS database, the KEGG and ChEBI databases (using the ChemSpider search node) and a Mass list with the molecules of interest (containing molecular formulas). A mass tolerance of 3 ppm was considered for all searches. The matches with the Mass list were further elucidated using the MS/MS data acquired and the results from the ChemSpider search, by performing FISh (Fragment Ion Search) scoring. This algorithm compares the experimental fragmentation spectra for a compound to the expected fragmentation spectra based on the structure of the compound, giving it a score from 0 to 100.

### GLT8D1 structural models and comparisons

The AlphaFold2 structural model for GLT8D1 (AF-Q68CQ7-F1) was used to identify closest structural homologs in the Protein Data Bank (PDB) using Dali^27^. The top ten structures with similarity to the GLT8D1 AlphaFold model were used for structure-based protein sequence alignment using PROMALS3D^28^. Protein structures were aligned and displayed using Pymol (Schrödinger, L., & DeLano, W. (2020). PyMOL. Retrieved from http://www.pymol.org/pymol).

### Supplementary Information

Supplementary Information.

## Data Availability

Data included in this work are available from the corresponding author upon reasonable request.

## Abbreviations

Endo H: Endoglycosidase H
Gal: Galactose
GalA: Galacturonic acid
GalNAc: *N*-Acetylgalactosamine
Glc: Glucose
GlcA: Glucuronic acid
GlcNAc: *N*-Acetylglucosamine
GFP: Green fluorescent protein
GT: Glycosyltransferase
GLT8D1: Glycosyltransferase 8 domain-containing protein 1
HPAEC-PAD: High-performance anion-exchange chromatography with pulsed amperometric detection
ITF-DSF: Dye-free differential scanning fluorimetry monitoring intrinsic tryptophan fluorescence
LC–MS: Liquid chromatography–mass spectrometry
LacNAc: *N*-Acetyllactosamine
PNGase F: Peptide *N*-Glycosidase F
*T*_*m*_: Melting temperature
Xyl: Xylose

## References

[1] Morais VA, Costa MT, Costa J (2003). *N*-Glycosylation of recombinant human fucosyltransferase III is required for its in vivo folding in mammalian and insect cells. Biochim. Biophys. Acta.

[2] Moremen KW (2018). Expression system for structural and functional studies of human glycosylation enzymes. Nat. Chem. Biol..

[3] Moremen KW, Tiemeyer M, Nairn AV (2012). Vertebrate protein glycosylation: Diversity, synthesis and function. Nat. Rev. Mol. Cell Biol..

[4] Reily C, Stewart TJ, Renfrow MB, Novak J (2019). Glycosylation in health and disease. Nat. Rev. Nephrol..

[5] Freeze HH, Eklund EA, Ng BG, Patterson MC (2015). Neurological aspects of human glycosylation disorders. Annu. Rev. Neurosci..

[6] Moremen KW, Haltiwanger RS (2019). Emerging structural insights into glycosyltransferase-mediated synthesis of glycans. Nat. Chem. Biol..

[7] Lairson LL, Henrissat B, Davies GJ, Withers SG (2008). Glycosyltransferases: Structures, functions, and mechanisms. Annu. Rev. Biochem..

[8] Taujale R (2021). Mapping the glycosyltransferase fold landscape using interpretable deep learning. Nat. Commun..

[9] Cooper-Knock J (2019). Mutations in the glycosyltransferase domain of GLT8D1 are associated with familial amyotrophic lateral sclerosis. Cell Rep..

[10] Wu Y (2020). Multi-trait analysis for genome-wide association study of five psychiatric disorders. Transl. Psychiatry.

[11] Yang CP (2018). Comprehensive integrative analyses identify GLT8D1 and CSNK2B as schizophrenia risk genes. Nat. Commun..

[12] Beddok A (2021). Germinal GLT8D1, GATAD2A and SLC25A39 mutations in a patient with a glomangiopericytal tumor and five different sarcomas over a 10-year period. Sci. Rep..

[13] Hu H (2019). GLT8D1 overexpression as a novel prognostic biomarker in human cutaneous melanoma. Melanoma Res..

[14] Xu H (2023). Glycosyltransferase GLT8D1 and GLT8D2 serve as potential prognostic biomarkers correlated with Tumor Immunity in Gastric Cancer. BMC Med. Genom..

[15] Ilina EI (2022). Enzymatic activity of glycosyltransferase GLT8D1 promotes human glioblastoma cell migration. iScience.

[16] Moll, T. *et al. BioRxiv*. 10.1101/2022.06.28.497990.

[17] Lombard V, Golaconda Ramulu H, Drula E, Coutinho PM, Henrissat B (2014). The carbohydrate-active enzymes database (CAZy) in 2013. Nucleic Acids Res..

[18] Sethi MK (2010). Identification of glycosyltransferase 8 family members as xylosyltransferases acting on O-glucosylated notch epidermal growth factor repeats. J. Biol. Chem..

[19] Tsai PC (2021). Genetic and functional analysis of glycosyltransferase 8 domain-containing protein 1 in Taiwanese patients with amyotrophic lateral sclerosis. Neurol. Genet..

[20] Qasba PK, Ramakrishnan B, Boeggeman E (2008). Structure and function of beta -1,4-galactosyltransferase. Curr. Drug Targets.

[21] Jumper J (2021). Highly accurate protein structure prediction with AlphaFold. Nature.

[22] Taujale R (2020). Deep evolutionary analysis reveals the design principles of fold A glycosyltransferases. Elife..

[23] Clarke BR (2020). A bifunctional O-antigen polymerase structure reveals a new glycosyltransferase family. Nat. Chem. Biol..

[24] Jiang YL (2017). Defining the enzymatic pathway for polymorphic O-glycosylation of the pneumococcal serine-rich repeat protein PsrP. J. Biol. Chem..

[25] Katz M, Diskin R (2022). Structural basis for matriglycan synthesis by the LARGE1 dual glycosyltransferase. PLoS One.

[26] Ramakrishnan B, Ramasamy V, Qasba PK (2006). Structural snapshots of beta-1,4-galactosyltransferase-I along the kinetic pathway. J. Mol. Biol..

[27] Holm L, Rosenstrom P (2010). Dali server: Conservation mapping in 3D. Nucleic Acids Res..

[28] Pei J, Kim BH, Grishin NV (2008). PROMALS3D: A tool for multiple protein sequence and structure alignments. Nucleic Acids Res..

[29] Yu H (2015). Notch-modifying xylosyltransferase structures support an SNi-like retaining mechanism. Nat. Chem. Biol..

[30] Gibbons BJ, Roach PJ, Hurley TD (2002). Crystal structure of the autocatalytic initiator of glycogen biosynthesis, glycogenin. J. Mol. Biol..

[31] Roversi P (2017). Interdomain conformational flexibility underpins the activity of UGGT, the eukaryotic glycoprotein secretion checkpoint. Proc. Natl. Acad. Sci. USA.

[32] Jorgensen R, Pesnot T, Lee HJ, Palcic MM, Wagner GK (2013). Base-modified donor analogues reveal novel dynamic features of a glycosyltransferase. J. Biol. Chem..

[33] Zhang Y, Xiang Y, Van Etten JL, Rossmann MG (2007). Structure and function of a chlorella virus-encoded glycosyltransferase. Structure.

[34] Culbertson AT, Ehrlich JJ, Choe JY, Honzatko RB, Zabotina OA (2018). Structure of xyloglucan xylosyltransferase 1 reveals simple steric rules that define biological patterns of xyloglucan polymers. Proc. Natl. Acad. Sci. USA.

[35] Alfaro JA (2008). ABO(H) blood group A and B glycosyltransferases recognize substrate via specific conformational changes. J. Biol. Chem..

[36] Chen R (2009). Glycoproteomics analysis of human liver tissue by combination of multiple enzyme digestion and hydrazide chemistry. J. Proteome Res..

[37] Latousakis D (2019). Serine-rich repeat protein adhesins from *Lactobacillus reuteri* display strain specific glycosylation profiles. Glycobiology.

[38] Sheikh MO (2017). Rapid screening of sugar-nucleotide donor specificities of putative glycosyltransferases. Glycobiology.

[39] Engel L, Alves J, Hennek J, Goueli SA, Zegzouti H (2021). Utility of bioluminescent homogeneous nucleotide detection assays in measuring activities of nucleotide-sugar dependent glycosyltransferases and studying their inhibitors. Molecules..

[40] de Vries T (1995). Acceptor specificity of different length constructs of human recombinant alpha 1,3/4-fucosyltransferases. Replacement of the stem region and the transmembrane domain of fucosyltransferase V by protein A results in an enzyme with GDP-fucose hydrolyzing activity. J. Biol. Chem..

[41] Sousa VL, Costa MT, Palma AS, Enguita F, Costa J (2001). Localization, purification and specificity of the full-length membrane-bound form of human recombinant alpha 1,3/4-fucosyltransferase from BHK-21B cells. Biochem. J..

[42] Sonnino S (2023). The relationship between depletion of brain GM1 ganglioside and Parkinson's disease. FEBS Open Bio..

[43] Piazzesi A, Afsar SY, van Echten-Deckert G (2021). Sphingolipid metabolism in the development and progression of cancer: One cancer's help is another's hindrance. Mol. Oncol..

[44] Brockhausen I, Melamed J (2021). Mucins as anti-cancer targets: Perspectives of the glycobiologist. Glycoconj. J..

[45] Williams SE (2022). Mammalian brain glycoproteins exhibit diminished glycan complexity compared to other tissues. Nat. Commun..

[46] Wilkinson H (2021). The O-glycome of human nigrostriatal tissue and its alteration in Parkinson's Disease. J. Proteome Res..

[47] Costa J, Hayes C, Lisacek F (2023). Protein glycosylation and glycoinformatics for novel biomarker discovery in neurodegenerative diseases. Ageing Res. Rev..

[48] Finne J (1975). Structure of the O-glycosidically linked carbohydrate units of rat brain glycoproteins. Biochim. Biophys. Acta.

[49] Gerloff DL (2022). Prediction and verification of glycosyltransferase activity by bioinformatics analysis and protein engineering. STAR Protoc..

[50] Kadirvelraj R (2018). Human *N*-acetylglucosaminyltransferase II substrate recognition uses a modular architecture that includes a convergent exosite. Proc. Natl. Acad. Sci. USA.

[51] Meng L (2013). Enzymatic basis for *N*-glycan sialylation: Structure of rat alpha2,6-sialyltransferase (ST6GAL1) reveals conserved and unique features for glycan sialylation. J. Biol. Chem..

[52] Boruah BM (2020). Characterizing human alpha-1,6-fucosyltransferase (FUT8) substrate specificity and structural similarities with related fucosyltransferases. J. Biol. Chem..

[53] Machado E (2011). *N*-Glycosylation of total cellular glycoproteins from the human ovarian carcinoma SKOV3 cell line and of recombinantly expressed human erythropoietin. Glycobiology.

[54] Costa J (2018). *N*-Glycosylation of extracellular vesicles from HEK-293 and glioma cell lines. Anal. Chem..

