# Supplementary material for: Glycosyltransferase 8 domain-containing protein 1 (GLT8D1) is a UDP-dependent galactosyltransferase
Source: Sci Rep. 2023 Dec 7;13:21684. doi: 10.1038/s41598-023-48605-4 (PMC10709319; doi:10.1038/s41598-023-48605-4)
Supplement: Supplementary file 1 — Supplementary Information. [file 41598_2023_48605_MOESM1_ESM.pdf]

Table S1. Saccharides tested in the enzymatic assays.

**Saccharides**	**Abbreviation**	**Reference**
Galactose	Gal	Carl-Roth 4987.1
N-Acetylglucosamine	GlcNAc	Carl-Roth, 8993.2
N-Acetylgalactosamine	GalNAc	Carl-Roth, 4114.2
Xylose	Xyl	Sigma PHR2102
Glucuronic acid	GlcA	Sigma G5269
N-acetylneuraminic acid	Neu5Ac	Dextra N01, Calbiochem CAS 3063-71-6
N-Acetylactosamine	LacNAc	Dextra GN204
Lacto-N-biose	GlcNAcβ1-3GlcNAc	Dextra GN203
di-N-acetylchitobiose	GlcNAcβ1-4GlcNAc	TCI D421520MG
Lewis^{a}	Galβ3(Fucα4)GlcNAc	Dextra LN304
Lewis^{x}	Galβ4(Fucα3)GlcNAc	Dextra LN303
Lacto-N-tetraose	Galβ3GlcNAcβ3Galβ4Glc	Dextra L403
Lacto-N-neotetraose	Galβ4GlcNAcβ3Galβ4Glc	Dextra L404
Lacto-N-fucopentaose I	Fucα2Galβ3GlcNAcβ3Galβ4Glc	Dextra L502
Lacto-N-fucopentaose II	Galβ3(Fucα4)GlcNAcβ3Galβ4Glc	Dextra L503
Biotinylated N-acetylglucosamineβ-$OCH_2CH_2NH_2$	GlcNAc-C2	Lectinity 0029a-BM

Table S2. N-Glycans tested in the enzymatic assays.

Sample ID	Designation	Structure SNFG	Molecular mass	Reference (Theraproteins)
#1	Asialylated agalactosylated biantennary glycan with bisecting GlcNAc and core fucose	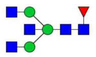	1665.62	GTP 0N-2A-2G+bGN+F
#2	Asialylated agalactosylated biantennary glycan with core fucose	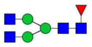	1462.54	GTP 0N-2A-2G+F
#3	Asialylated agalactosylated biantennary glycan	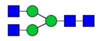	1316.49	GTP 0N-2A-2G
#4	Asialylated biantennary glycan with bisecting GlcNAc and core fucose	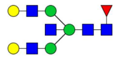	1989.73	GTP 0N-2A+bGN+F
#5	Asialylated monogalactosylated biantennary glycan with bisecting GlcNAc and core fucose	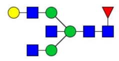	1827.68	GTP 0N-2A-1G+bGN+F (C6)
#6	Asialylated agalactosylated biantennary glycan with bisecting GlcNAc	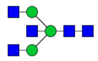	1519.57	GTP 0N-2A-2G+bGN
#7	Asialylated monogalactosylated biantennary glycan with core fucose	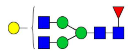	1624.6	GTP 0N-2A-1G+F (C3/C6)
#8	Asialylated monogalactosylated biantennary glycan	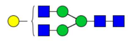	1478.54	GTP 0N-2A-1G (C3/C6)
#9	Asialylated agalactosylated monoantennary glycan with core fucose	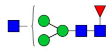	1259.47	GTP 0N-2A-2G-1GN+F (C3/C6)
#10	Asialylated agalactosylated monoantennary glycan	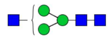	1113.41	GTP 0N-2A-2G-1GN (C3/C6)
#11	Asialylated biantennary glycan with core fucose	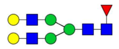	1786.65	GTP 0N-2A+F
#12	Asialylated biantennary glycan	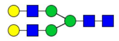	1640.59	GTP 0N-2A
#13	Asialylated triantennary glycan with core fucose	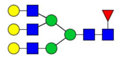	2151.78	GTP 0N-3A(2,6)+F
#14	Asialylated agalactosylated triantennary glycan with core fucose	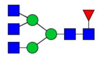	1665.62	GTP 0N-3A(2,6)-3G+F
#15	Asialylated tetraantennary glycan with core fucose	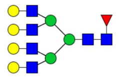	2516.91	GTP 0N-4A+F
#16	Asialylated tetraantennary glycan	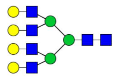	2370.86	GTP 0N-4A

Table S3. Proteins with structural similarity to GLT8D1 AlphaFold model based on a DALI search of the PDB database[a]

CAZy family	**Enzyme**	**Species**	**Enzyme donor**	**PDB code**	**Mechanism**	***Dali* Z score**	**Sequence identity[a] (%)**	**RMSD (Å)**	**Aligned residues[b]**
GT8	WbbM	*Klebsiella pneumoniae*	UDP-Gal	6U4B	retaining	24.2	20	7	263
GT8	GlyE	*Streptococcus pneumoniae*	UDP-Gal	5GVV	unknown[c]	22.7	21	2.5	222
GT8	LARGE-1	*Homo sapiens*	UDP-Xyl	7UI7	retaining	21.7	16	3.6	251
GT24	UGGT	*Chaetomium thermophilum*	UDP-Glc	5N2J	retaining	21.5	15	8.5	253
GT8	GT8 protein	*Anaerococcus prevotii*	Unk	3TZT	unknown[c]	19.7	16	2.6	211
GT8	Glycogenin-1	*Oryctolagus cuniculus*	UDP-Glc	1LL0	retaining	18.7	20	3.4	224
GT8	Glycogenin-1	*Homo sapiens*	UDP-Glc	8CVZ	retaining	18.2	18	3.1	221
GT8	XXYLT1	*Mus musculus*	UDP-Xyl	4WLG	retaining	17.4	16	3.6	232
GT6	ABO	*Homo sapiens*	UDP-Gal/GalNAc	3V0Q	retaining	14.3	7	4.2	223
GT34	UDP-Glc GlcT	*Chorella virus*	UDP-Glc	2P73	unknown[c]	11.4	15	3.4	178
GT34	XXT1	*Arabidpopsis thaliana*	UDP-Xyl	6BSU	retaining	11.2	8	3.7	203

[a] The full-length AlphaFold model of GLT8D1 was used to search the PDB database using Dali [22] to identify proteins with structural similarity. Numerous proteins were identified, including multiple hits for different PDB files from the same protein. The sequence identity, rmsd value and residues aligned were obtained using the Dali Pairwise server.
[b] Number of structurally equivalent residues.
[c] Mechanism has not been identified.

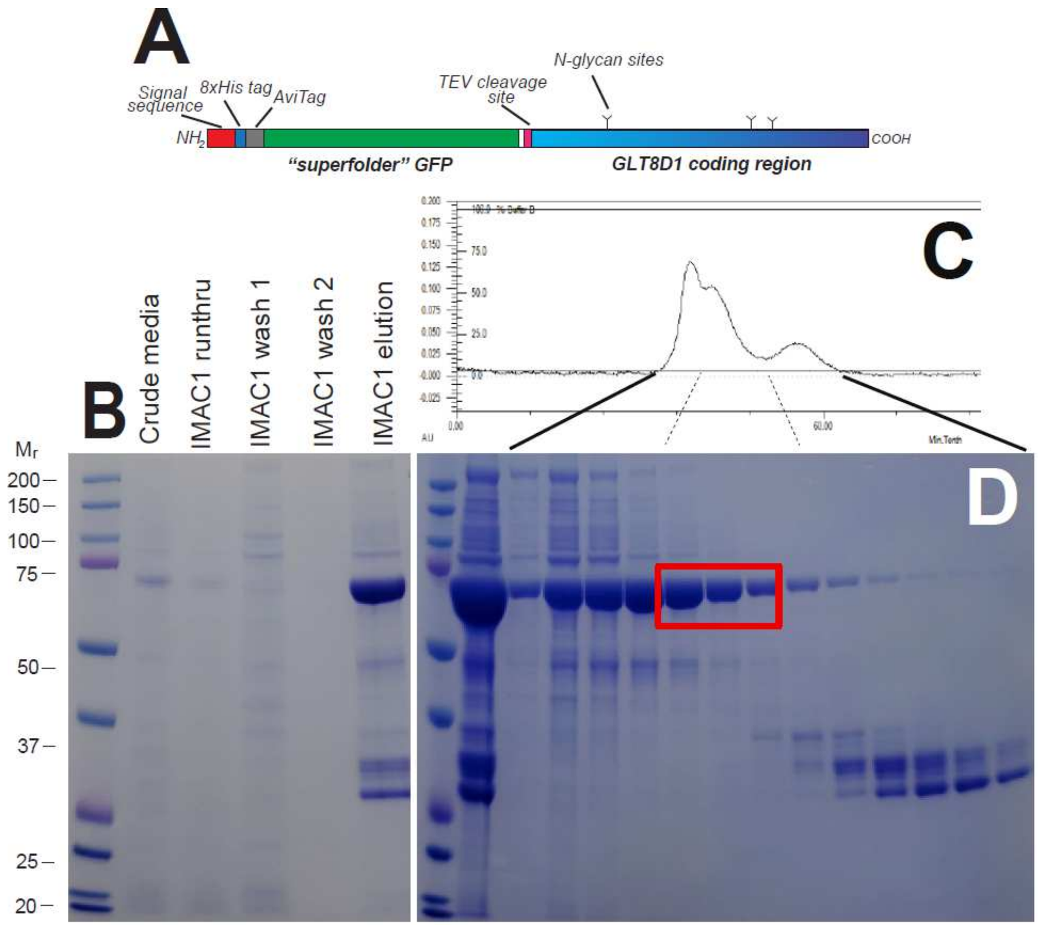

Fig. S1. Expression of human GLT8D1 in mammalian cells and purification as a GFP fusion protein. Panel A, cartoon representation of the fusion protein assembly for expression in mammalian cells. Transfection and production in HEK293 cells leads to the secretion of a fusion protein containing an N-terminal 8xHis tag, an AviTag, GFP domain, and TEV protease cleavage site, followed by the truncated ectodomain of GLT8D1. The three N-glycans are shown in stick representation. Panel B, the conditioned media from the expression culture (crude media) was passed through a Ni2+-NTA column (IMAC1 run-through) and washed (IMAC1 wash 1 and 2) before elution with 300 mM imidazole (IMAC1 elution). The sample was concentrated and resolved on a Superdex 75 gel filtration column (Panel C) and individual fractions were run on a SDS-PAGE gel (Panel D). Fractions representing purified GLT8D1 were pooled (red box) and represent 3 mg of purified protein from one liter of expression culture.

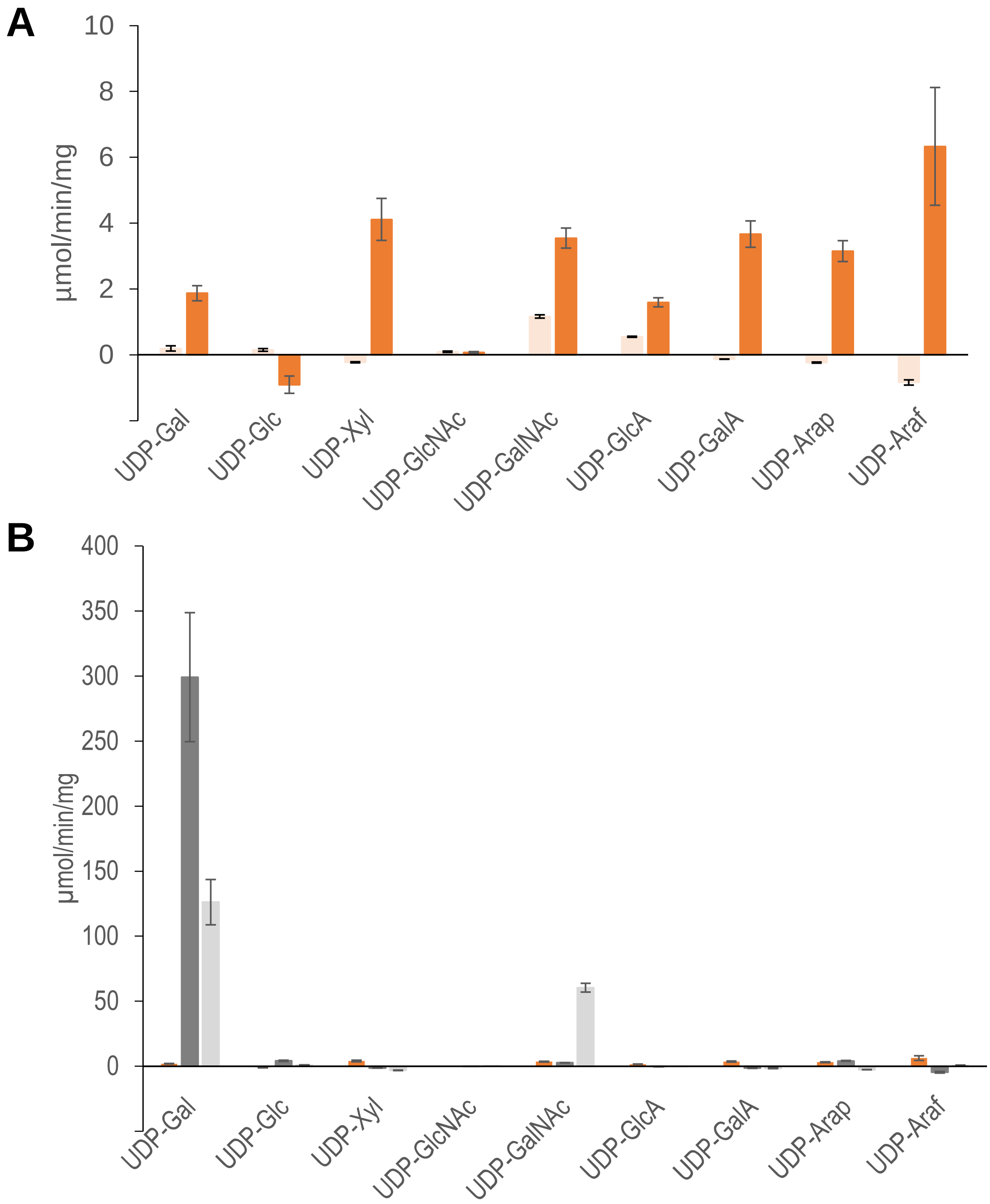

Fig. S2. Hydrolytic activity of GLT8D1 towards UDP-based donor nucleotide sugars. (A) Activity in the presence (dark orange) or absence of 10 mM Mn^{2+} (light orange). (B) Activity of GLT8D1 (dark orange) compared with control B4GALT1-WT (dark grey) and B4GALT1-Y289L (light grey). Assays were done in duplicate. The values presented correspond to the activity observed subtracted by the control without enzyme. Some values are negative, which is due to variations among the assays; they become evident since the activity values are very low confounding with the background and the number of replicates are also low. Thus, negative values have no physical significance.

Name	Formula	Annot. Source	Annot. ΔMass [ppm]	Calc. MW	RT [min]	Area (Max.)	# ChemSpider Results	# mzCloud Results	mzCloud Best Match	mzCloud Best Match Confidence	Mass List Matches	MS2
Gal-GalNAc	C14 H25 N O11		-0.23	383.14267	5.171	26350347	80	2	77.4	28.9		

Structure	Name	Formula	Molecular Weight	FISh Coverage
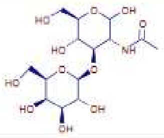	Lacto-N-biose I	C14 H25 N O11	383.14276	76.67
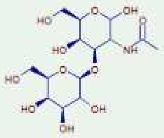	beta-D-Galp-(1->3)-D-GalpNAc	C14 H25 N O11	383.14276	76.67
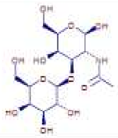	T ANTIGEN	C14 H25 N O11	383.14276	70.00
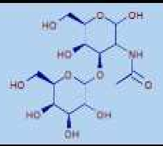	alpha-D-Galp-(1->3)-alpha-D-GalpNAc	C14 H25 N O11	383.14276	70.00

Fig. S3. MS2 Fish Score results for all ChemSpider (KEGG DB) entries. Compounds with top FISh coverage matching (3 ppm mass tolerance) the MS2 spectrum of GLT8D1 product with UDP-Gal and GalNAc as substrates (Figure 5). MS2 spectra are presented in the next page.

Sample_Pos_01_CE30_5ul (F12) #2059, RT=5.130 min, MS2, FTMS (+), (HCD, DDA, 384.1499@30, +1)
Lacto-N-biose I, C14 H25 N O11
FISh Coverage: 12 Matched, 4 Unmatched, 6 Skipped

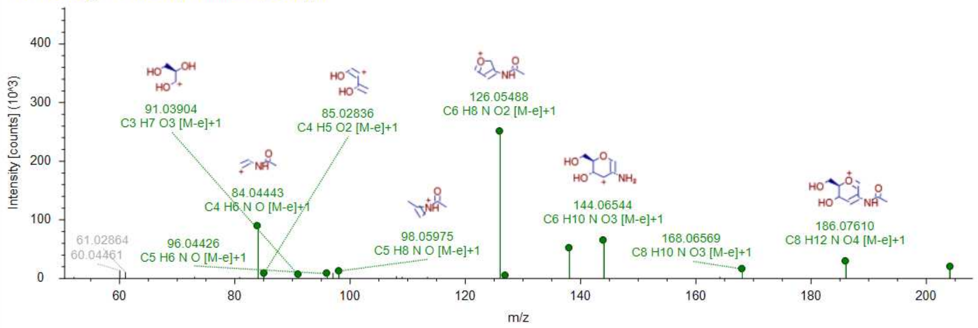

Sample_Pos_01_CE30_5ul (F12) #2059, RT=5.130 min, MS2, FTMS (+), (HCD, DDA, 384.1499@30, +1)
beta-D-Galp-(1->3)-D-GalpNAc, C14 H25 N O11
FISh Coverage: 12 Matched, 4 Unmatched, 6 Skipped

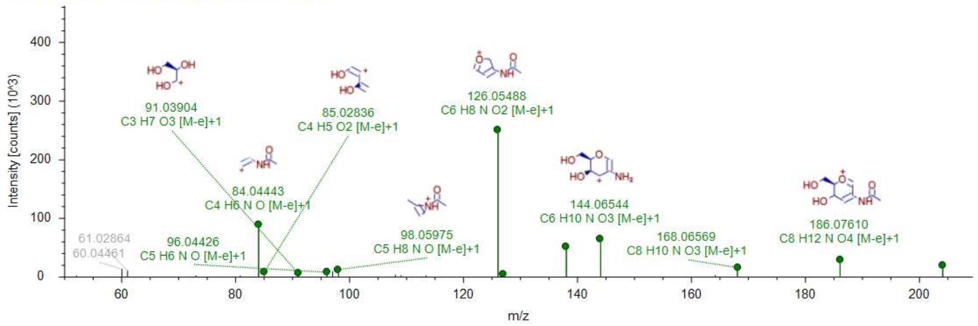

Sample_Pos_01_CE30_5ul (F12) #2059, RT=5.130 min, MS2, FTMS (+), (HCD, DDA, 384.1499@30, +1)
T ANTIGEN, C14 H25 N O11
FISh Coverage: 11 Matched, 5 Unmatched, 6 Skipped

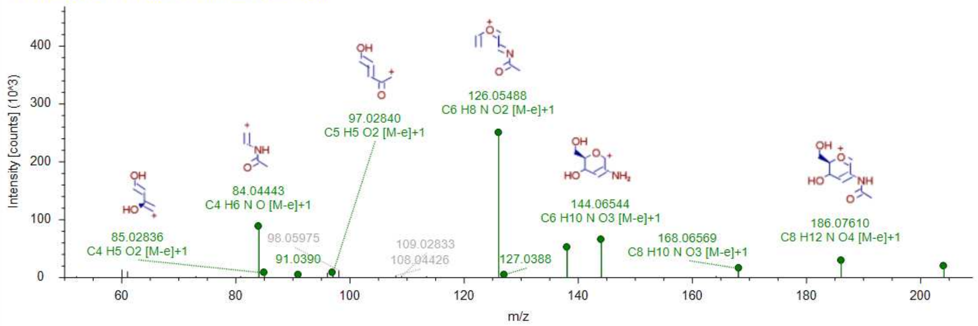

Sample_Pos_01_CE30_5ul (F12) #2059, RT=5.130 min, MS2, FTMS (+), (HCD, DDA, 384.1499@30, +1)
alpha-D-Galp-(1->3)-alpha-D-GalpNAc, C14 H25 N O11
FISh Coverage: 11 Matched, 5 Unmatched, 6 Skipped

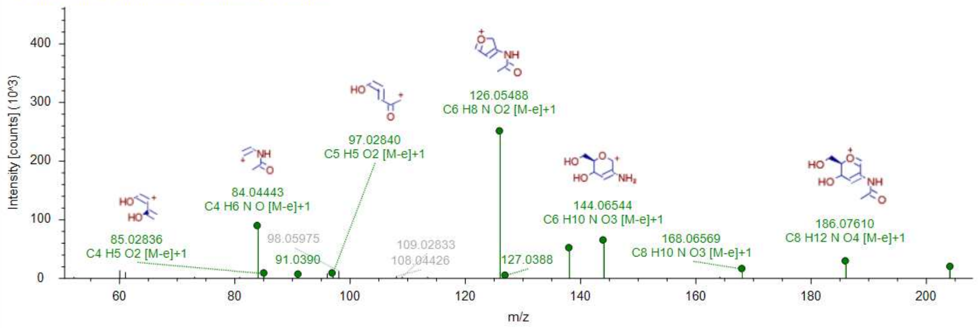

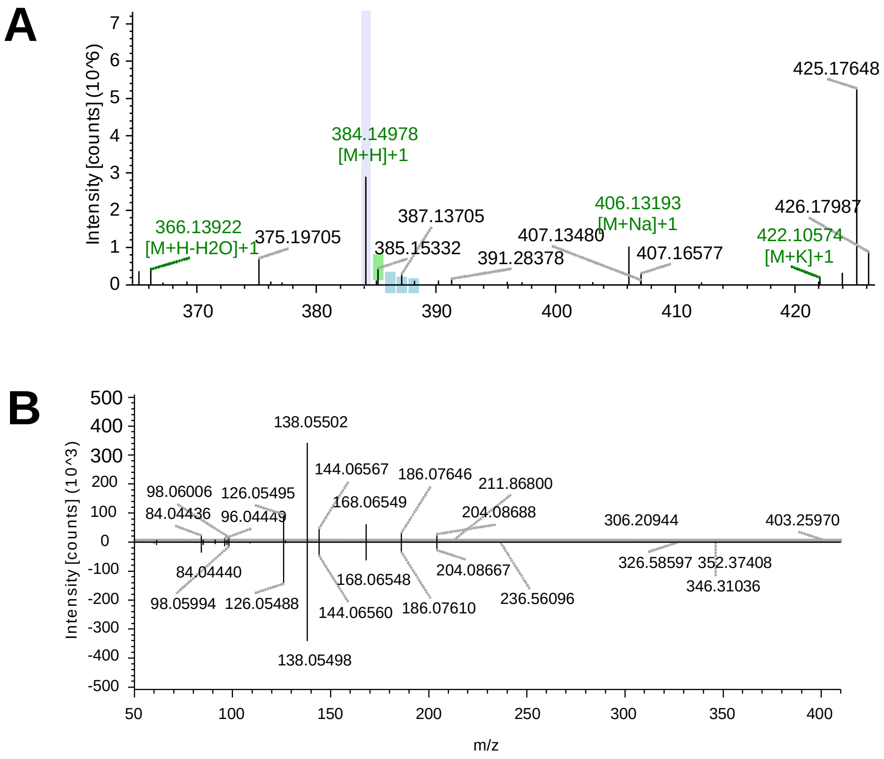

Fig. S4. LC-MS/MS analysis of the reaction product of GLT8D1 using UDP-Gal as nucleotide sugar donor and GlcNAc as acceptor.

(A) Peak at *m/z* 384.15 corresponding to the composition HexHexNAc is shown.

(B) Comparison of MS^2 spectrum of reaction product with that of standard LacNAc (Galβ4GlcNAc) analyzed in similar conditions (98.2% match).

A

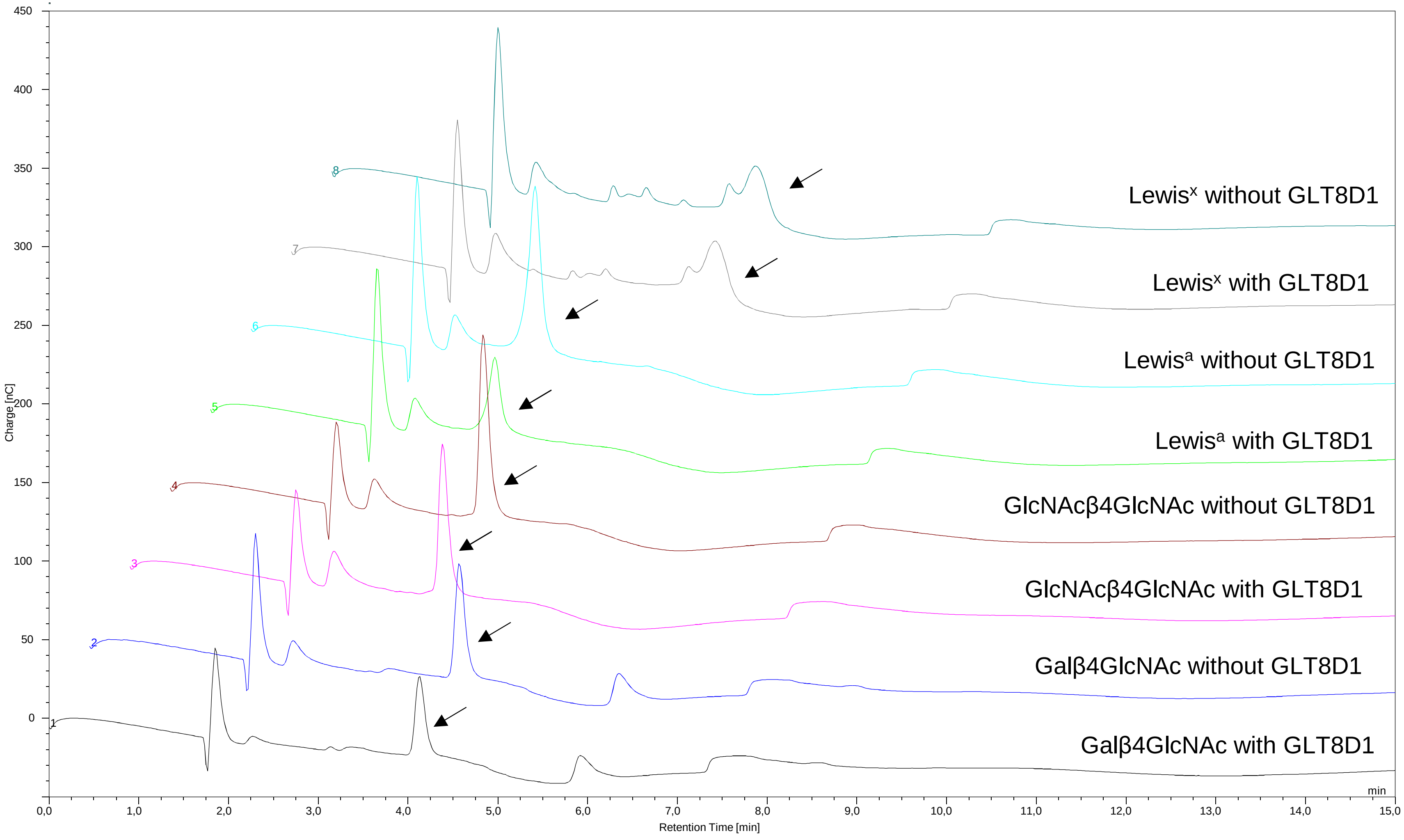

Fig. S5. HPAEC PAD chromatograms of oligosaccharides (see Table S1) incubated with GLT8D1. The profiles of the control assay where GLT8D1 was replaced by the corresponding buffer are shown for comparison. The peaks corresponding to the indicated oligosaccharides are pointed with arrows.

A) Di- and trisaccharides.

B) Tetra- and pentasaccharides (next page).

B

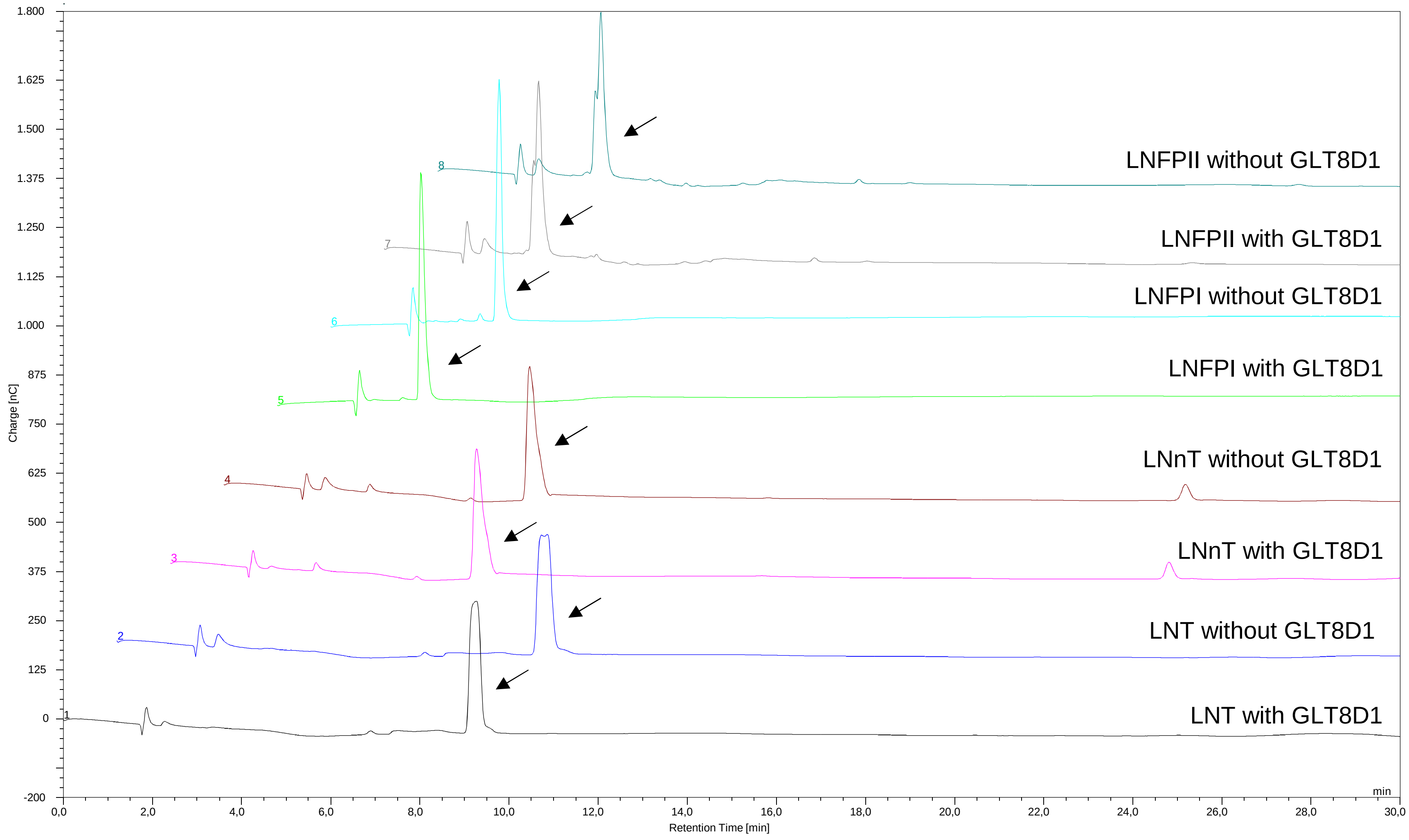

1.800
1.625
1.500
1.375
1.250
1.125
1.000
875
750
625
500
375
250
125
0
-200
Charge [nC]
0,0
2,0
4,0
6,0
8,0
10,0
12,0
14,0
16,0
18,0
20,0
22,0
24,0
26,0
28,0
30,0
min
Retention Time [min]
1
2
3
4
5
6
7
8
LNFPII without GLT8D1
LNFPII with GLT8D1
LNFPI without GLT8D1
LNFPI with GLT8D1
LNnT without GLT8D1
LNnT with GLT8D1
LNT without GLT8D1
LNT with GLT8D1

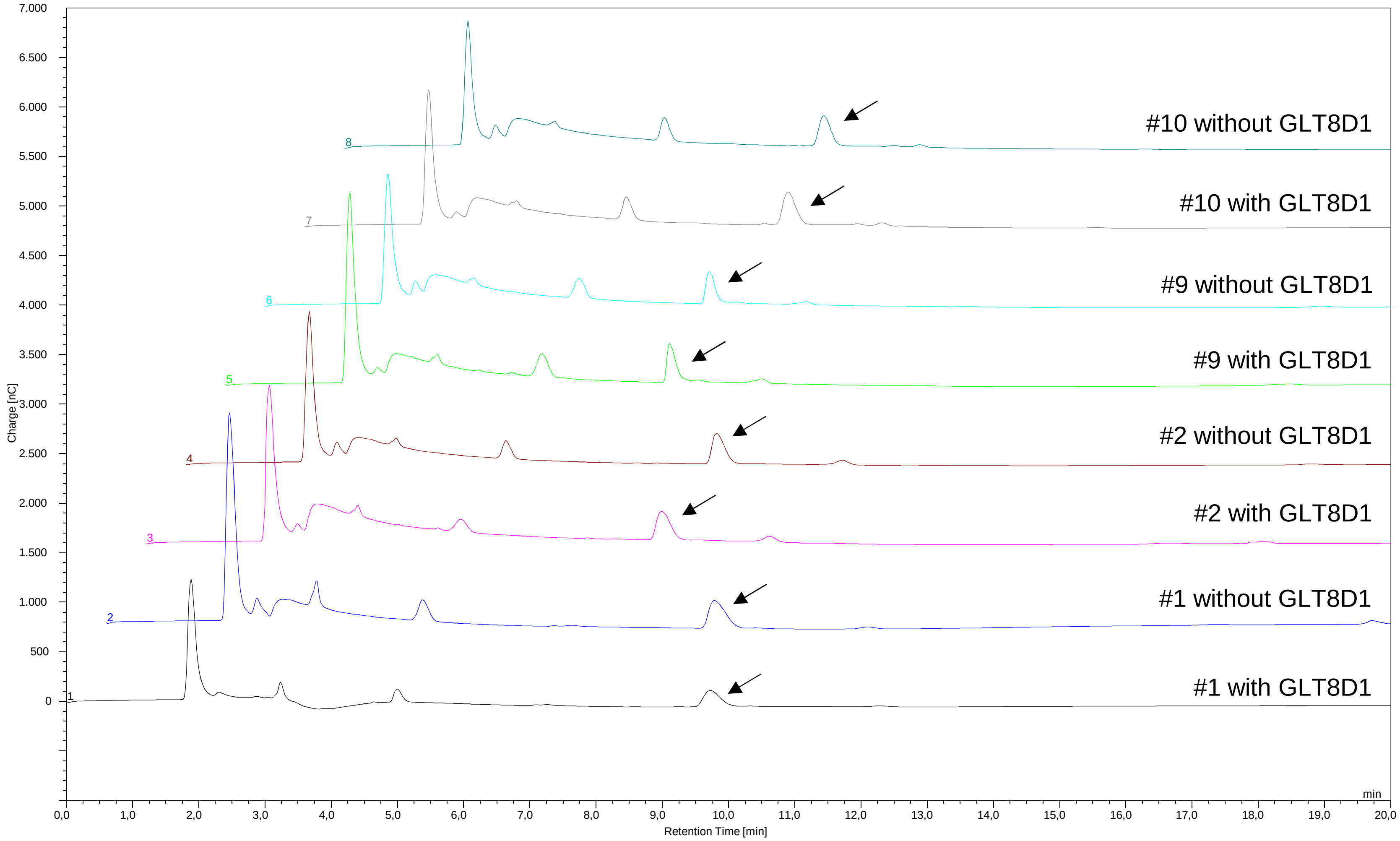

Fig. S6. HPAEC PAD chromatograms of N-glycans incubated with GLT8D1. The profiles of the non-treated standard and the control assay where GLT8D1 was replaced by the corresponding buffer were shown. The profiles with or without GLT8D1 were similar, which indicated that these compounds were not efficient enzyme substrates. The peaks corresponding to the indicated N-glycans are pointed with arrows.

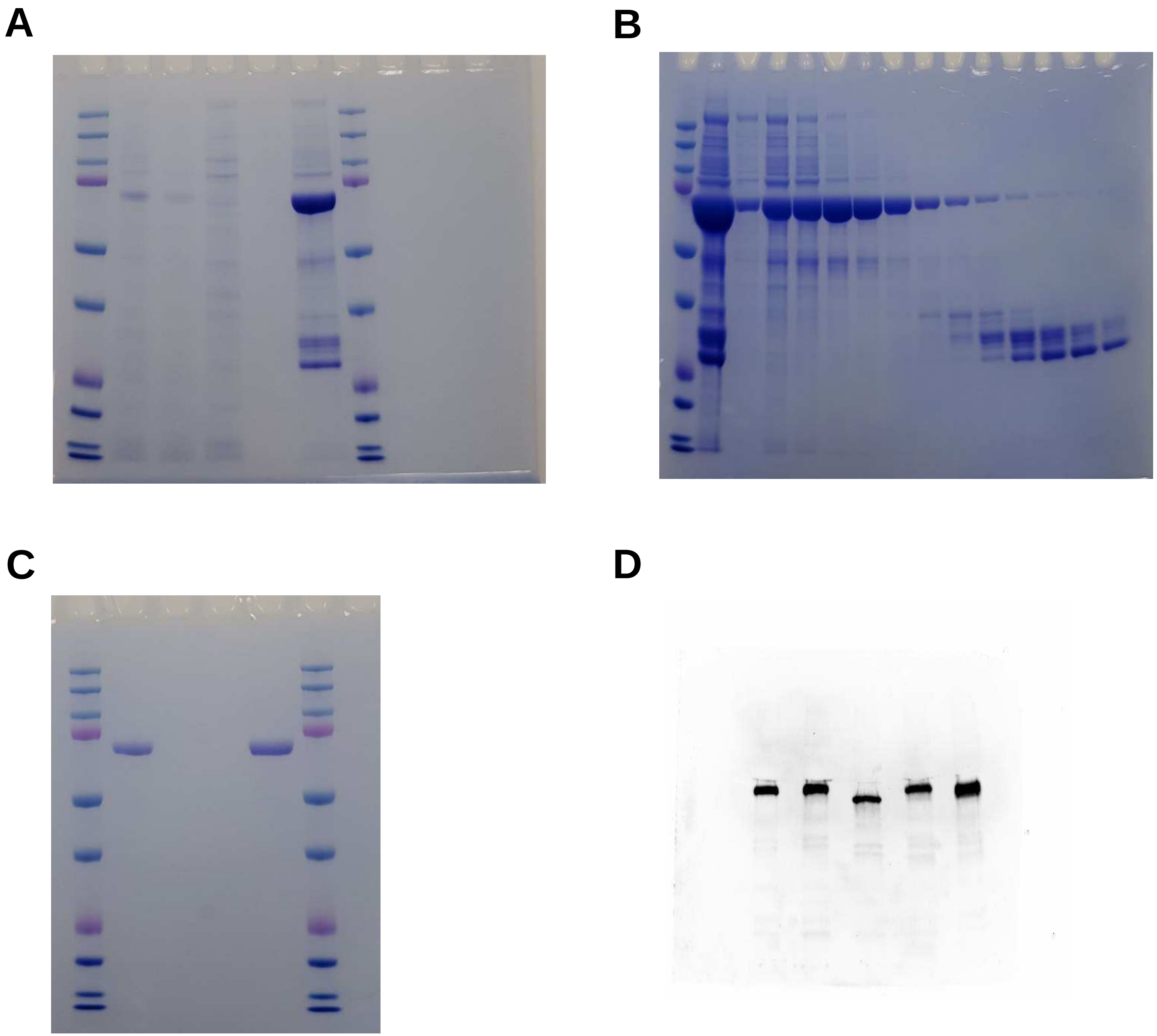

Fig. S7. Original images corresponding to Fig. S1 B (A), Fig. S1 D (B), Fig. 1B (C) and Fig. 1C (D).
